# Development of a composite scoring system to rank communities at high risk of zero-dose children in Cameroon: A geospatial analysis

**DOI:** 10.7189/jogh.13.04136

**Published:** 2023-11-17

**Authors:** Yauba Saidu, Valirie N Agbor, Pietro Di Mattei, Sangwe C Nchinjoh, Nnang N Edwidge, Andreas A Njoh, Nkwain J Muteh, Marta Prescott, Owens Wiwa, Demba Diack, Junie Flegere, Emanuele Montomoli, Sue A Costa Clemens, Ralf Clemens

**Affiliations:** 1 Clinton Health Access Initiative Inc., Yaoundé, Cameroon; 2 Institute for Global Health, Department of Molecular and Developmental Medicine, University of Siena, Siena, Italy; 3 Clinical Trial Service Unit and Epidemiological Studies Unit (CTSU), Nuffield Department of Population Health, University of Oxford, Oxford, UK; 4 Gavi, the Vaccine Alliance, Geneva, Switzerland; 5 Expanded Program on Immunization, Cameroon Ministry of Public Health, Yaoundé, Cameroon; 6 School of Global Health and Bioethics, Euclid University, Bangui, Central African Republic; 7 Global Analytics and Implementation Research Team, Clinton Health Access Initiative Inc., Boston, USA; 8 VisMederi srl, Siena, Italy; 9 Department of Pediatrics, University of Oxford, Oxford, UK; 10 International Vaccine Institute, IVI, Seoul, Republic of Korea

## Abstract

**Background:**

Despite growing efforts to improve access to vaccination, millions of children, especially in developing countries, have not received a single dose of diphtheria, tetanus, and pertussis (DTP) vaccine. Consequently, they are often called zero-dose children (ZDC). With limited health resources, prioritising communities for rapid and mass zero-dose catch-up vaccination in missed communities to avert epidemic outbreaks is complicated by unreliable denominators used to compute vaccination coverages. Incorporating other indicators of access and utilisation of vaccination services can help with identifying and ranking missed communities based on the likelihood of finding ZDC. We described the process of generating a scoring method to rank health areas in Cameroon based on their likelihood of containing ZDC.

**Methods:**

We used geospatial analysis to compute and aggregate health area characteristics, including hard-to-reach (HTR) areas (defined as areas of settlement above a one- (for urban areas) or 15-kilometre radius (for rural areas) beyond a vaccinating health facility), amount of area covered by slums and new area settlement, and percentage of children unvaccinated for DTP-1. We attributed a weight based on the ability to limit accessibility or utilisation of vaccination services to each characteristic and computed the score as a weighted average of health area characteristics. The health area score ranged from 0 to 1, with higher scores representing a higher likelihood of containing ZDC. We stratified the analysis by rural and urban health areas.

**Results:**

We observed substantial district and regional variations in health area scores, with hotspots health areas (administrative level 4) observed in the Far North (0.83), North (0.81), Adamawa (0.80), East (0.75), and South West (0.67) regions. The Adamawa region had the highest percentage of health areas with the highest score (78%), followed by the East (50%), West (48%), and North (46%) regions. For most regions (Far North, South, South West, Littoral, West, and North West), DTP-1 contributed the most to the score. However, HTR settlement areas within a health area contributed substantially to the overall score in the East, North, and Adamawa regions.

**Conclusions:**

We found substantial variations in health area scores with hotspots in the Far North, North, Adamawa, East, and South West regions. Although DTP-1 could be used as an indicator to identify health areas with ZDC for most communities, HTR settlement area was a valuable indicator in ranking priority health areas in the East, North, and Adamawa regions, further emphasising the need to consider other indicators before prioritisation.

Immunisation is crucial for preventing maternal and child disability and death, especially in low- and middle-income countries where infectious diseases are endemic [1]. The introduction of vaccines over the last decade has led to significant reductions in vaccine-preventable diseases, childhood disability, and death, and a reduction in health inequalities in several regions worldwide [2,3]. Globally, vaccination prevents approximately six million deaths and 96 million disability-adjusted life-years from vaccine-preventable diseases annually [4,5]. Additionally, high levels of population immunity (herd immunity) have led to the eradication of infectious diseases (where man is the only host of the causative agent) associated with high morbidity and mortality, such as smallpox [6], making it crucial for protecting vulnerable populations who, for some reason, cannot take a vaccine (e.g. severely ill individuals) or whose immune systems respond sub-optimally to vaccination (e.g. elderly people).

The Expanded Program on Immunisation (EPI) in Cameroon primarily seeks to prevent, control, or eradicate vaccine-preventable diseases in Cameroon [7], with a specific objective of attaining diphtheria, tetanus, and pertussis (DTP)-3 vaccination coverage of at least 90% at the national level and 80% at the district level. Consequently, EPI combined its activities into the minimum package of activities of health facilities nationwide following the Reorientation of Primary Health Care in 1993 and recommended key vaccination strategies for routine implementation by health facilities, such as fixed vaccination sessions, outreach vaccination for distant communities, and temporarily fixed post-vaccination strategy [7,8]. Despite these efforts, vaccination coverage remains unacceptably low in some health districts, leading to an overall below-target national coverage. In fact, the number of Cameroon's under-vaccinated and zero-dose children (ZDC) has been rising in recent years. An analysis of data for 2018-2019 from Cameroon's District Health Information Software 2 (DHIS-2) showed that the percentage of infants who did not receive DTP-1 and DTP-3 increased by 6% (from 124 425 in 2018 to 131 818 in 2019) and 12% (from 189 336 in 2018 to 211 283 in 2019), respectively. Similarly, the number of infants who did not receive the first dose of the measles vaccine increased by about 4% (from 250 396 in 2018 to 258 763 in 2019). These numbers have increased dramatically with the COVID-19 pandemic [8,9]; the number of infants who did not receive DTP-3 rose by about 33%, from 171 453 in the second quartile of 2019 to 227 131 in the second quartile of 2020.

There have been limited efforts in identifying and reaching ZDC in Cameroon, defined as children who are eligible, but have never received a dose of the DTP-1 vaccine from their national EPI program. In 2019, the Clinton Health Access Initiative (CHAI), with Cameroon's EPI, surveyed six urban and one rural health districts in Cameroon to identify under-vaccinated and ZDC and enumerated 3767 households in 91 of the 358 existing slums in Yaoundé and 2753 children aged 0-24 months. The survey found 903 (33%) children above nine months of age who were under-vaccinated and 378 (14%) who were ZDC. Similarly, 854 (31%) ZDC in the rural district were identified in one fishing island. This experience suggests that there may be many more of such children in other missed communities in Cameroon, including slum dwellers in other cities, nomads, pygmies, islanders, and mountain people.

Currently, the percentage of children unvaccinated for DTP1 is used to define zero-dose communities. However, in settings like Cameroon, where the denominators used to compute vaccination coverages are unreliable, incorporating other indicators of access and utilisation of vaccination services can help with identifying missed communities. Here we describe the process of generating a score to rank health areas in Cameroon that are most likely to contain ZDC.

## METHODS

To generate the score, we aggregated selected characteristics at the health area level that indicate limited accessibility or utilisation of vaccination services. We then weighted health area characteristics to produce an average score for each health area, which we consider to be a proxy for the likelihood of finding ZDC in that health area. The weighting of these characteristics was based on their relative importance in preventing accessibility or use of vaccination or other related health care services.

### Indicators and sources of data

#### Amount of very hard-to-reach areas

We calculated the amount of very hard-to-reach areas using two variables.

The first variable was the geographical location of health facilities providing vaccination services. We obtained data on the different health facilities in Cameroon offering vaccination services and their geographical location from the DHIS-2 and the World Health Organisation (WHO) office in Cameroon. We defined health facilities providing vaccination services as those declared by the WHO to be providing these services or those that reported (in the DHIS-2) the number of children vaccinated with DPT1 between 1 January and 31 December 2020. Consequently, we assumed that health facilities that the WHO declared not providing vaccination services and had missing values for the number of children vaccinated with DPT1 in the DHIS-2 between 1 January and 31 December 2020 were not providing vaccination services.

The second variable used was the location of all settlements in Cameroon. We obtained the related data from Grid-3, which reports the location for three types of settlements: built-up areas (BUA), small settlement areas (SSA), and hamlets [10]. BUAs are urban areas with moderately-to-severely-spaced buildings, with visible grids of streets and blocks. They usually contain about 400 000m^2^ with a building density ≥30 across the entire area. SSAs are areas of permanently inhabited structures and compounds of a few hundred to a few thousand inhabitants. They are non-built-up areas with ≥50 buildings. Finally, hamlets comprise several compounds or sleeping houses in isolation from small settlements or urban areas and are characterised by low-density settlements between one and 50 buildings that fall within 65 metres of one another.

We defined hard-to-reach (HTR) settlements as the area of SSA or hamlet beyond a 15-kilometre radius of health facilities providing vaccination services. We used a 15-kilometre cut-off because this is well beyond the five-kilometre radius required for outreach vaccination activities. For urban areas (BUA), we defined HTR settlements as those beyond a one-kilometre radius around the closest health facilities providing vaccination services. We considered health areas with higher areas of settlement beyond the defined boundaries to be more likely to contain ZDC.

#### Amount of health areas covered by slums and new settlements

For Yaoundé, we also used the amount of a health area covered by slums or new settlements to proxy the likelihood of containing ZDC. Higher areas of slums and new settlements indicated a higher likelihood of finding ZDC. We obtained data on slums and new settlements from CHAI, Cameroon.

#### Health area diphtheria, pertussis, and tetanus (DPT) 1 non-vaccination rate

This referred to the percentage of children not vaccinated for DPT 1 in the health area in 2020. We obtained the DPT coverage rate for each health area in 2020 from DHIS-2 and used to calculate the percentage of unvaccinated children, as follows:

100% − *percentage of children vaccinated for DTP 1 in 2020*

A higher percentage of unvaccinated children for DPT-1 for a given health area indicated a higher likelihood of containing ZDC.

### Calculating the weighted score for ZDC at the health area level

After calculating the value of each indicator (i.e. HTR built-up areas, HTR small settlement areas, HTR hamlets, amount of area covered by slums and new area settlement, and percentage of children unvaccinated for DTP-1) per health area, we ranked the values into percentile groups (before weighting and calculating an overall score for each health area). For example, to create percentile ranks of the health area-specific values of the percentage of unvaccinated for DTP-1, we ordered the health areas from lowest to highest based on their value for this indicator and ranked them into ten groups (or percentiles). The lowest and highest percentile groups were attributed scores of 0.1 and 1.0, respectively. We repeated this process for each indicator. Using percentiles ensures that the indicators used to generate the score are on a standardised unit scale before combining them into a single summary score.

After ranking, we multiplied the health area ranked score for each indicator by the weight attributed to that indicator, which we in turn attributed based on the relative importance of the indicator in capturing accessibility or use of vaccination or other related primary health care services. The relative importance of these indicators was decided through discussions by researchers and national vaccination experts at CHAI and EPI Cameroon. We assigned a weight of three to the percentage of unvaccinated children with DPT-1 within a health area, a weight of two to the amount of extremely HTR SSA, hamlets, and BUA within a health area, and a weight of one to the amount of health areas covered by slums or new settlements.

We calculated the weighted score by taking a weighted average of the indicators included in the score. For example, we calculated the weighted score (r_zd_) for a given health area, using three indicators A, B, and C, with weights *wi*_A_, *wi*_B_, and *wi*_C_, respectively, as follows:

R_zd_ = ((percentile rank of A × *wi_A_*) + (percentile rank of B × *wi_B_*) + (percentile rank of C × *wi_C_*))/(*wi_A_* + *wi_B_* + *wi_C_*)

We calculated the score separately for rural (those with SSA and hamlets) and urban health areas (those with BUA). We considered health areas with higher scores as more likely to contain ZDC.

We performed all analyses using QGIS and R statistical programming, version 3.5.1 (R Core Team, Vienna, Austria).

## RESULTS

### Distribution health area scores

**Figure 1:**
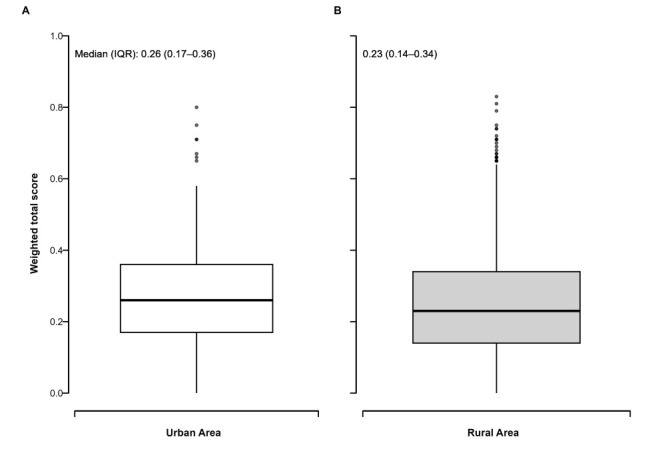
Distribution of weighted health area scores. **Panel A.** Urban health areas. **Panel B.** Rural health areas. IQR – interquartile range.

We found significant variations in scores in both urban (0.23 (interquartile range (IQR) = 0.14-0.34)) and rural health (0.26 (IQR = 0.17-0.36) areas (**Figure 1**, Panels A-B). However, these scores are not comparable, as they were generated using different methods. Among all rural and urban health areas, Waza and Ndelbe had the highest scores among all rural and urban health areas, respectively (**Table 1** and **Table 2**).

**Table 1:** Health areas with the highest and lowest scores

Area	Highest score	Lowest score
Rural	Waza (0.83)	Bamunkumbit (0), Bandongoue (0), Bengue Tiko (0), Bifang (0), Bimba (0), Eka (0), Ounangare Dimeo (0)
Urban	Ndelbe (0.8)	Akwa Nord (0), Bonassama (0), Famla 1 (0), Logbaba Centre (0), Messa (0), Nkololoun (0), Oyack 1 (0), Saint Michel (0)

**Table 2:** Health areas with highest and lowest scores per region

Region	Highest score	Lowest score
Adamawa	Ndelbe (0.8)	Tchabal Mbabo (0.05), Atta (0.05)
Centre	Nyamoko (0.66)	Messa (0)
East	Belabo (0.75)	Bandongoue (0), Bengue Tiko (0), Bimba (0)
Far North	Waza (0.83)	Ounangare Dimeo (0)
Littoral	Yingui (0.66)	Akwa Nord (0), Bonassama (0), Logbaba Centre (0), Nkololoun (0), Oyack 1 (0), Saint Michel (0)
North	Mbang-Rey (0.81)	Babla (0.01), Pologozom (0.01)
North West	Buku (0.66)	Bamunkumbit (0), Bifang (0), Eka (0)
South	Londji (0.61)	Melondo (0.01)
South West	Kajifu (0.67)	Big Ngbandi (0.01), Bangem (0.01), Ebamut (0.01)
West	Manda (0.56)	Famla 1 (0)

### Geographical distribution of health areas scores

**Figure 2:**
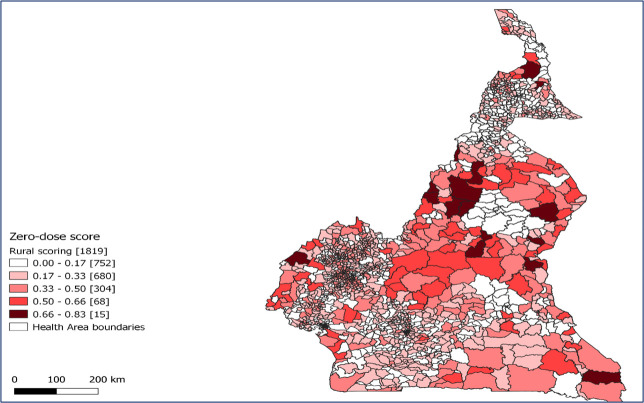
Geographical distribution of health area scores showing locations with highest to lowest probability of finding ZDC. Darker shades correspond to higher probability.

Figure 2 shows health areas where ZDC are likely to be concentrated. ZDC seems to be concentrated in health areas in most regions in Cameroon; however, hotspots in health areas can be observed in the Far North, North, Adamawa, East, and South-West regions.

### Trends in health area scores

**Figure 3:**
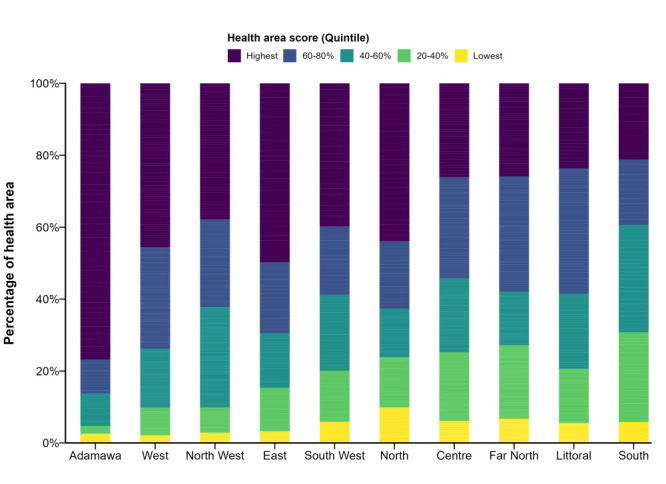
Trends in health area scores in the Regions of Cameroon. Each color represents the quintiles of health area scores for each region.

When ranked by quintiles according to percentages, health areas in the highest fifth are most likely to contain ZDC, while those in the lowest fifth are least likely to contain ZDC. For example, Adamawa region had the highest percentage of health areas (78%) within the highest quintile, followed by the East (50%), West (47%), and North (42%) regions (**Figure 3**).

### Contribution of the indicators to the final score

**Figure 4:**
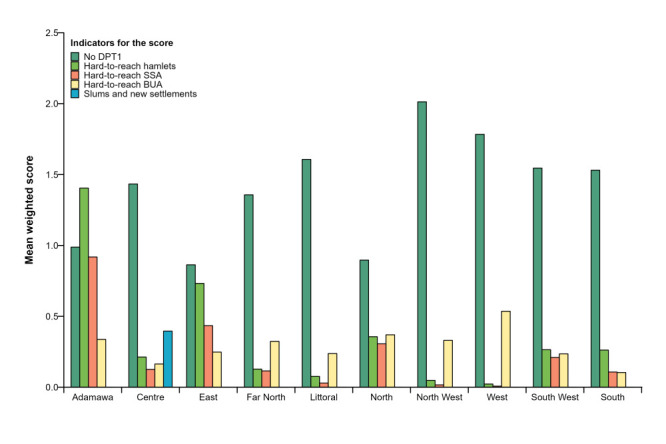
Mean weighted score of each indicator by region. DPT1 – first dose of the diphtheria-pertussis-tetanus vaccine. BUA – built-up areas. SSA – small settlement areas.

We ranked each indicator by quartile and then applied weights and plotted the mean weighted score for each indicator per region (**Figure 4**). For most regions (Far North, South, South West, Littoral, West and North West), DTP-1 contributed the most to the score. However, areas of HTR hamlets and small settlement areas significantly contributed to the overall score in the East, North, and Adamawa regions.

## DISCUSSION

ZDC and missed communities still exist, despite significant advances in vaccination coverage. According to the 2018 Cameroon Demographic and Health Survey report, only about 53% of children between 12 and 23 months had received all essential vaccines, including a dose of Bacillus Calmette-Guerin (BCG) and measles vaccines, and three doses of polio and DTP vaccine [11]. Children in rural areas (45%) were less likely to have received all essential vaccines than those in urban areas (61%). Moreover, about 10% of children 12-23 months old had not received any essential vaccine; these cases are also known as ZDC [11]. Missed communities are more likely to experience disease outbreaks, which are associated with increased morbidity and mortality in affected children [12,13]. We described the process of generating a score to identify health areas that are likely to contain ZDC in Cameroon. We employed validated methods used in previous studies to produce quantile estimates for each health area [14].

We observed substantial variations between health areas, districts, and regions in health area scores, suggesting considerable equity challenges, even within districts and regions with overall high performance. This heterogeneity highlights the importance of performing analyses even on health area levels to minimise the financial, human, and material resources to identify ZDC and missed communities. Poor socioeconomic status, long distance to facilities providing vaccination services, and residence in rural areas are significant barriers to vaccination services [15]. In most regions, the percentage of children not vaccinated for DPT1 was the major driver of the zero-dose score, suggesting that DPT-1 could be a valid indicator for ZDC in most regions. However, other indicators (like HTR areas) contributed significantly to health area scores in the North, Adamawa, and East regions. This suggests that most settlements in these regions are distant from health facilities providing vaccination services and the importance of these indicators in prioritising local health areas. While outreach vaccination sessions and mass immunisation campaigns may capture most missed children in HTR communities, about 25% of these children are likely to be missed during these activities [16].

This approach to identifying health areas at risk of ZDC has potential implications for population health. The ranking of health areas can help the EPI prioritise the allocation of resources to areas at high risk of ZDC to improve vaccination coverage. Consequently, this strategy could ameliorate the national immunisation coverage, enable countries to attain herd immunity, and prevent outbreaks of and eradicate vaccine-preventable diseases. This method is also sustainable, as data analysts and statisticians at the central health level can be trained to develop this score – they can work with a multidisciplinary team of experts on vaccination to select and assign appropriate weights to each of the component items. This scoring approach is further generalisable to other settings, but the items used for the score and the weights attributed to each item must be decided by consulting local vaccination experts. Once the approach has been adopted, it might be helpful for immunisation programs to measure performance over multiple time periods to assess whether the identification of high-risk regions indeed leads to an increased performance of the regions

There are variables we deemed appropriate to include in the score, but were unavailable at the time of the analysis, such as data on the number of healthcare workers and the outbreak of a vaccine-preventable disease in the health area, which indicate limited availability and accessibility to vaccination services. Although we were aware of the different regions in conflict, data on the health areas affected by these conflicts was not available. Accessibility to cities is an indicator of socioeconomic inequality and access to health services such as vaccination. We considered this indicator relevant in the context of Cameroon, where vaccines are stored at the central level, with stable electricity, and then supplied to rural areas. By the time of this analysis, data on accessibility to cities provided by the Malaria Atlas Project was not available for Cameroon [17]. Moreover, we did not have data on social class, which is a determinant of access to vaccination and other primary health care services. Where available, it is important to consider including these indicators in the score. Additionally, updating information on the geo-location of health facilities providing vaccination services will improve the estimation of the amount of HTR areas of settlements. Finally, the predictive ability of this score to identify ZDC needs to be validated.

## CONCLUSION

We observed substantial variations in health area scores with hotspots in the Far North, North, Adamawa, East, and South-West regions. Besides the percentage of children unvaccinated for DTP1, the amount of HTR areas significantly contributed to the overall score, especially in regions like the Adamawa and East regions. In settings where data on the denominators to compute DPT1 coverages are unreliable, this analysis might be cost-effective in planning interventions to recover unvaccinated children.
